# Vinegar after an Operation

**Published:** 1898-02

**Authors:** 


It is said that if the patient is allowed to inhale vinegar after an operation, while coming out from the anesthesia, the nausea and vomiting will be prevented. Pour it on the mask or towel and let it be inhaled as ether is.—*Med. Council.*

